# Overlapping illusions by transformation optics without any negative refraction material

**DOI:** 10.1038/srep19130

**Published:** 2016-01-11

**Authors:** Fei Sun, Sailing He

**Affiliations:** 1 Centre for Optical and Electromagnetic Research, Zhejiang Provincial Key Laboratory for Sensing Technologies, JORCEP, East Building #5,Zijingang Campus, Zhejiang University, Hangzhou 310058, China; 2 Department of Electromagnetic Engineering, School of Electrical Engineering, Royal Institute of Technology (KTH), S-10044 Stockholm, Sweden

## Abstract

A novel method to achieve an overlapping illusion without any negative refraction index material is introduced with the help of the optic-null medium (ONM) designed by an extremely stretching spatial transformation. Unlike the previous methods to achieve such an optical illusion by transformation optics (TO), our method can achieve a power combination and reshape the radiation pattern at the same time. Unlike the overlapping illusion with some negative refraction index material, our method is not sensitive to the loss of the materials. Other advantages over existing methods are discussed. Numerical simulations are given to verify the performance of the proposed devices.

Transformation optics (TO) is a powerful tool to design electromagnetic/optical devices with pre-designed function to achieve an unprecedented controlling of the light wave^1^,^2^,^3^. Many novel devices have been designed or even experimentally demonstrated by TO including invisibility cloaking^1^,^4^,^5^,^6^, perfect imaging device^7^,^8^, power combination^9^,^10^,^11^,^12^,^13^,^14^, radiation suppression^15^ and other optical illusions^16^.

The perfect lens, composed by the negative refraction index materials (NIMs)^17^, can be treated as a space folding transformation from the perspective of transformation optics (TO)^2^. Owing to the space folding transformation, a single point in the reference space may correspond to many points in the real space (e.g., folding for one time will produce three points in the real space corresponding to one single point in the reference space). These points in the real space are the equivalent points (i.e. they all corresponds to the same point in the reference space). If we set one point source at any one of these equivalent points, we will obtain the images at the rest of these equivalent points. Based on this idea, some novel optical devices that can create the overlapping effect of the light sources have been proposed for the power combinations when the coherent light sources are in-phase in these equivalent points^9^,^10^,^11^,^12^,^13^, or the radiation suppression when two coherent light sources located at two equivalent points are out of phase^15^. The function of these devices is to shift the radiation pattern of a light source inside the device to a free space region outside the device, and to achieve an overlapping illusion when the radiation patterns of many such light sources are shifted to one common location in the free space (i.e. to achieve a power combination effect). To achieve such an overlapping illusion, both space folding transformation and the space compression transformation are required (i.e. the NIMs are required in these devices), and the transformation medium designed by this method is often referred as an optical illusion medium^9^,^10^ or a shifting medium^11^,^13^.

The overlapping illusion designed by the space folding transformation that involves the NIMs is greatly influenced by the loss of the materials to achieve a negative refraction index effect. Actually there are many other ways to make many different points in the real space equivalent (i.e. they corresponds to the same single point in the reference space from the perspective of TO) in addition to the space folding transformation. Generally speaking, any non-monotonic (i.e. multiple-valued) coordinate transformations can lead to such an effect, e.g. an angle scaling transformation, an extremely stretching spatial transformation, some special conformal mapping^14^, etc. In this paper, we use an extremely stretching spatial transformation to create an overlapping illusion without any NIMs. After some calculations, we found that only one homogenous anisotropic medium (referred as the optic-null medium (ONM)) is needed to create such an optical overlapping illusion.

## The optic-null medium (ONM)

The optic-null medium (ONM) is a homogenous block of highly anisotropic medium designed by the extremely stretching spatial transformation^18^,^19^,^20^,^21^,^22^,^23^,^24^,^25^,^26^,^27^. The ONM has been applied in many different applications, e.g. an optical hyper-lens^18^,^23^,^24^, DC magnetic hose^25^, concentrators^26^. We also introduced the idea of further transformations inside an ONM to reduce the material requirement of devices designed by TO^20^,^27^, and extended the theory of TO to the optical surface transformation (OST) by the ONM^21^,^22^. The ONM has been experimentally demonstrated by metamaterials at microwave frequencies^18^,^19^,^23^.

**Figure 1:**
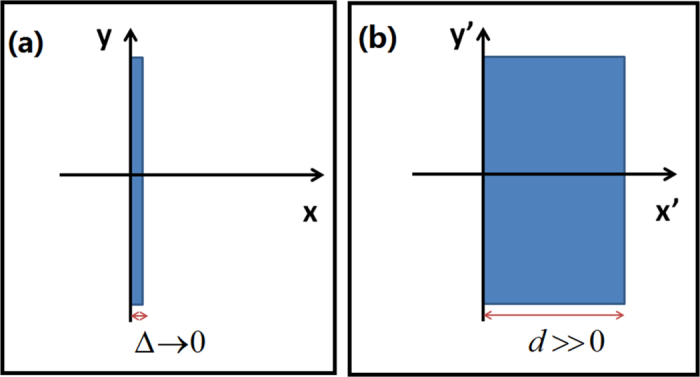
(**a**) The reference space: a very thin volume (Δ→0) that can approximately treated as a surface whose normal vector is along x direction. (**b**) The real space: the ONM whose main axis is along *x*′ direction. A surface in the reference space is extremely stretched to a volume filled with the ONM in the real space. The main axis of the ONM is in accordance with the direction of the stretching. In this paper, we assume the quantities with or without the upper primes indicate the quantities in the real or reference space, respectively.

As shown in Fig. 1, an ONM with main axis along *x*′ direction can be obtained from extremely stretching an extremely thin volume (i.e. it can approximately be treated as a surface whose normal vector is along *x* direction) along *x* direction in the reference space. The coordinate transformation can be given as:


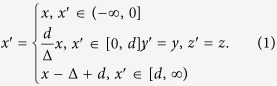


With the help of TO^1^,^2^,^3^, we can calculate the relative permittivity and permeability of the ONM related to the above coordinate transformation:


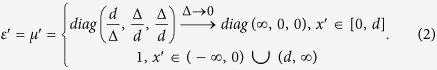


From the above calculations, we can see that an ONM (i.e. the medium filling the region *x*′∈[0, *d*]) with the main axis along the *x*′ direction can be obtained with an extreme stretching of the space along this direction. Indeed, this can be done in any other directions (e.g. along the radial direction). One important feature of an ONM can be concluded from the above coordinate transformation: since the thin blue volume filled with the ONM in the real space (see Fig. 1(b)) is obtained by stretching a thin blue surface in the reference space (see Fig. 1(a)), all points along the same line (e.g. *y*′ = Constant and *z*′ = Constant) inside the ONM are equivalent points that correspond to the same point in the reference space. Therefore we can use these equivalent points to achieve an overlapping illusion.

## Overlapping illusion by an ONM

**Figure 2:**
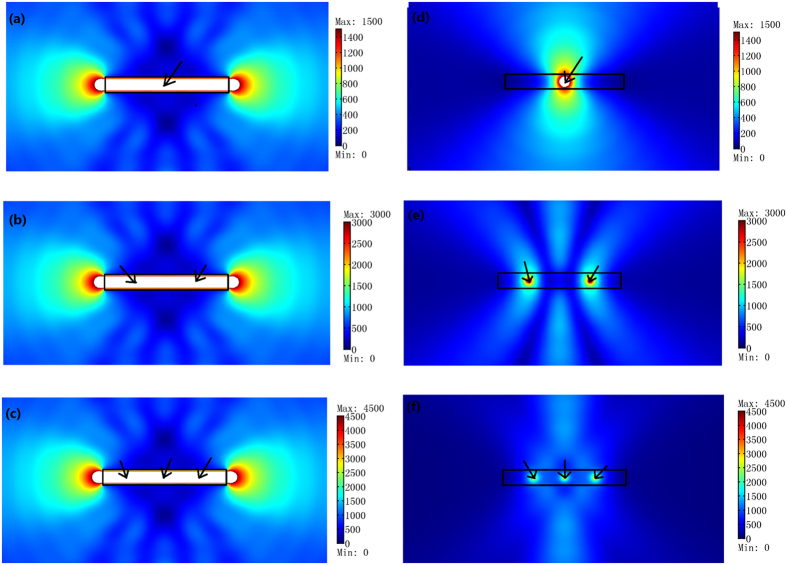
The 2D finite element method (FEM) simulation results. We plot the absolute value of the *z*′ component of the electric field for the TE wave case. The rectangular ONM with the main axis along the *x*′ direction has a length *d* = 8λ_0_/3 along *x*′ direction and height *h* = 2λ_0_/3 along *y*′ direction. (**a**) Only one line current with unit amplitude 1A is set at the center of the rectangular ONM. (**b,c**) are two and three in-phase coherent line currents with unit amplitude set in the equivalent positions (i.e. in the same line *y*′ = Constant), respectively. The black arrows indicate the locations of the line currents. For comparison, we also simulate the case that the ONM is removed from (**a–c**), which correspond to (**d–f**).

The overlapping illusion created by an ONM is shown in Fig. 2. We use the COMSOL Multiphysics to make numerical simulations in the paper. We should note that from Fig. 2(a–c), the absolute values of the electric field’s distributions are exactly the same, while the color bars are magnified by one, two, and three times. If the number of the coherent in-phase sources is *N*, the total radiation power produced by the whole system is *N*^2^*I*_0_, where *I*_0_ is the power produced by a single source. This is due to the fact that these line currents are set at the equivalent positions, and hence the total field in the whole space is always a maximum interference if these line currents are in-phase, which is called as a perfect coherent effect^11^,^28^.

**Figure 3:**
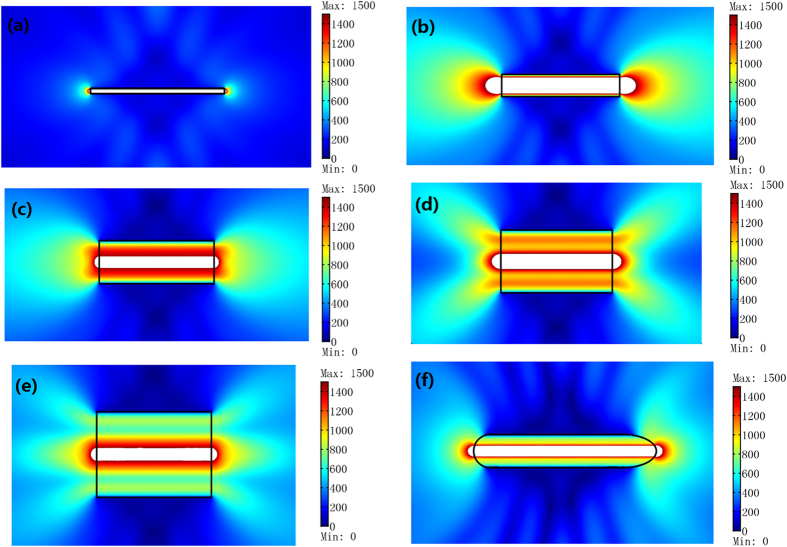
The absolute value of the *z*′ component of the electric field for a TE polarization by the 2D FEM simulations. The rectangular ONM with the main axis along the *x*′ direction has a length *d* = 8λ_0_/3 along *x*′ direction and height *h* varying in (**a–e**). (**a**) *h* = 0.1λ_0_, (**b**) *h* = 0.5λ_0_, (**c**) *h* = λ_0_, (**d**) *h* = 1.5λ_0_, and (**e**) *h* = 2λ_0_. (**f**) The ONM with a circular and an elliptical shaped surfaces. The white region means the value is beyond the maxima in the color bar. We set a line current with unit amplitude in the center of the ONM in all cases.

We should note that unlike previous designs to achieve an optical overlapping illusion^9^,^10^,^11^,^12^,^13^,^14^, the ONM can achieve an overlapping illusion and a radiation pattern reshaping effect at the same time. As shown in Fig. 2(a–c), the radiation pattern is no longer a cylindrical wave (like Fig. 2(d)) but some other shaped pattern. Furthermore, the final radiation pattern of the composited light sources can be tailored by changing the size or the shape of the ONM we used (see Fig. 3).

**Figure 4:**
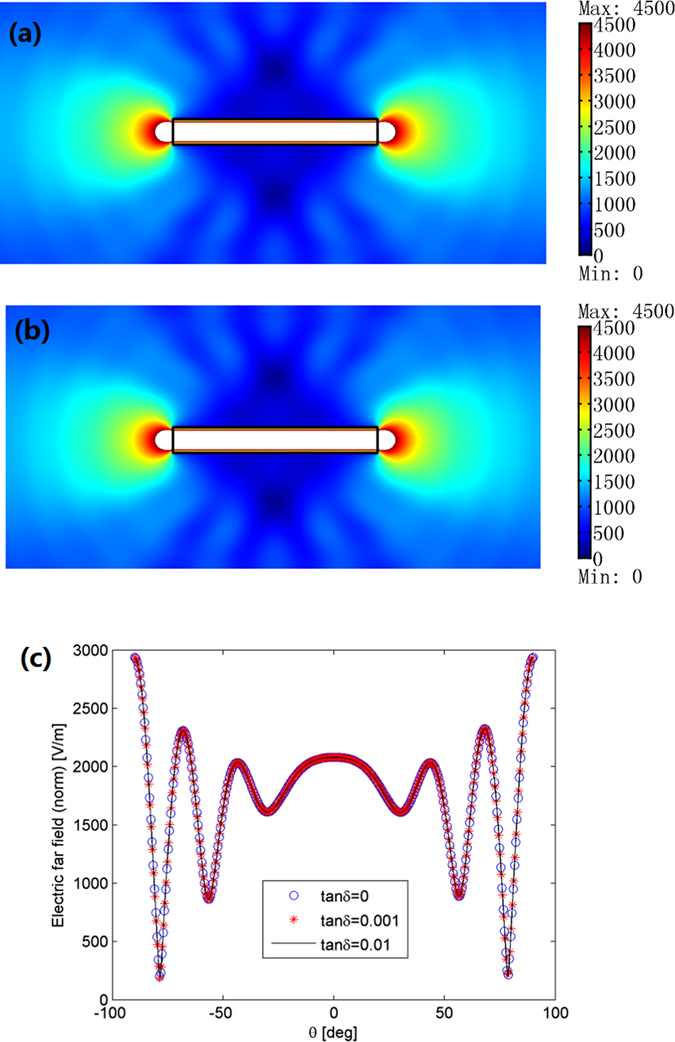
The performance of the ONM for overlapping illusion if some loss is introduced. The size of the ONM and the distribution of line currents are the same as the those in Fig. 2(c). (**a,b**) show the absolute value of the *z*′ component of the electric field when a loss tangent *δ* = 0.001 and *δ* = 0.01 is added to each material parameter, respectively. The field almost has no changes with Fig. 2(c). (**c**) The far field patterns for different loss tangents are introduced.

Next we study the performance of the ONM if some loss is introduced. As shown in Fig. 4(a,b), the radiation patterns remain almost the same as Fig. 2(c) even after a small loss is introduced. From the far field radiation pattern in Fig. 4(c), we can see the performance of the ONM for overlapping illusion is not sensitive to the material loss.

## The radiation suppression

**Figure 5:**
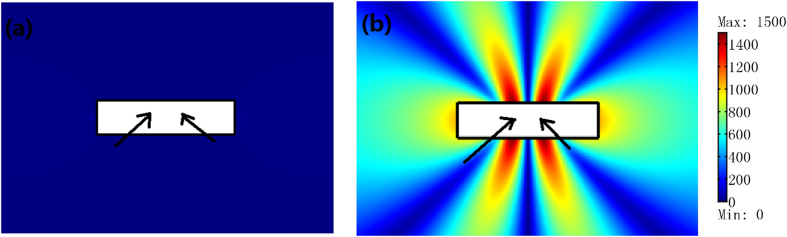
The performance of the ONM for the radiation suppression by a 2D FEM simulation. We plot the absolute value of the *z*′ component of the electric field for the TE polarization. We set two coherent line currents with the same unit amplitude but out of phase as indicated by the black arrows. (**a**) the white region is filled with the ONM whose main axis is along *x*′ direction. (**b**) the white region is filled with air.

In addition to the power combination, the ONM can also be utilized to achieve the radiation suppression. As shown in Fig. 5(a), the radiation is greatly suppressed if we set two coherent line currents out of phase at the equivalent points inside the ONM (compared with the case when we remove the ONM in Fig. 5(b)).

## Conclusion and Discussion

Compared with the overlapping effect by other methods^9^,^10^,^11^,^12^,^13^,^14^, the overlapping illusion created by the ONM in this paper has many special features. For the overlapping illusion by NIMs, we need more separated shifting devices to achieve a higher power^9^,^10^,^11^,^12^,^13^. However, we can only use a single device composed by the ONM to achieve a higher power by simply adding more separated light sources inside the device (see Fig. 2). Furthermore the overlapping illusion created by the ONM is not sensitive to the loss (see Fig. 4), while the performance of an illusion device composed by NIMs is sensitive to the loss (especially in the far field)^12^.

The overlapping illusion devices designed by the conformal mapping are inhomogeneous (e.g. a gradient control is needed), which makes it hard to realize these devices^14^. However, the ONM is a homogenous medium that can also give the overlapping illusion. The number of the equivalent sources that can be utilized to the power combination should be determined before designing the illusion device by the optical conformal mappings, which means that if the number of the light sources changes, one has to find some other suitable mapping to redesign the device^14^. For the power combination by the present ONM, we do not have such a problem and actually we have unlimited equivalent locations.

In addition to the above points, the ONM can produce not only an overlapping illusion but also a radiation pattern reshaping effect together (see Fig. 3). This means that we can achieve a power combination with a desired radiation pattern at the same time by a single device composed by the ONM.

The perfect coherence can also be achieved by zero-index material (ZIM) (i.e. both mu and epsilon are nearly zero)^29^. The geometry of ZIM can also be used to reshape the radiation pattern of the overlapped power. The overlapping illusion produced by a ZIM and an ONM are different: For a ZIM, all components of the permittivity and permeability are nearly zero; for an ONM the components of the permittivity and permeability in the directions orthogonal to its main axis are nearly zero, while the permittivity and permeability along its main axis are extremely large. If we replace the ONM in Fig. 2 by a ZIM, no such phenomenon would appear. The overlapping illusion produced by an ONM can be explained from the perspective of TO: there are infinite equivalent points inside the ONM, and hence an overlapping illusion will appear if we set many sources at these equivalent points (i.e. they all correspond to the same point in the reference space). The proposed method in this paper may have potential applications in perfect coherence, power combination, radiation pattern control, and radiation suppression, etc.

Another note we want to make is how to realize such an ONM. The ONM is a highly anisotropic medium whose relative permittivity and permeability are very large in its main axis direction (e.g. we took 1000 in simulations) and nearly zero in other orthogonal directions (e.g. we took 0.001 in simulations). Actually there have been some experimental demonstrations on the realization of such an ONM^18^,^19^,^23^. He *et al.* chose a holey metallic plate structure to realize such an ONM that performs like an optical hyperlens^18^. Another way to realize such an ONM is to use a metallic slit array, which has also been experimentally demonstrated for a concentrator^19^. Sadeghi *et al.* experimentally demonstrated a metallic slit array that satisfies the Fabry-Pérot resonance can perform equivalently like an ONM. Their experimental result has also shown that the loss in the metallic slit array doesn’t influence the performance of the ONM^19^.

## Additional Information

**How to cite this article**: Sun, F. and He, S. Overlapping illusions by transformation optics without any negative refraction material. *Sci. Rep.* **6**, 19130; doi: 10.1038/srep19130 (2016).

## References

[b1] Pendry J. B., Schurig D. & Smith D. R. Controlling electromagnetic fields. Science 312, 1780–1782 (2006). 16728597 10.1126/science.1125907

[b2] Chen H., Chan C. T. & Sheng P. Transformation optics and metamaterials. Nat. Mater. 9(5), 387–396 (2010). 20414221 10.1038/nmat2743

[b3] Werner D. H. & Kwon D. H. Transformation Electromagnetics and Metamaterials: Fundamental Principles and Applications (London:Springer-Verlag, 2014).

[b4] Schurig D. *et al.* Metamaterial electromagnetic cloak at microwave frequencies. Science 314(5801), 977–980 (2006). 17053110 10.1126/science.1133628

[b5] Chen H. & Zheng B. Broadband polygonal invisibility cloak for visible light. Sci. Rep. 2, 255 (2012). 22355767 10.1038/srep00255 PMC3275922

[b6] Sun F. & He S. A third way to cloak an object: cover-up with a background object Prog. Electromagn. Res. 149, 173–182 (2014).

[b7] Wang W. *et al.* Design of oblate cylindrical perfect lens using coordinate transformation. Opt. Express 16(11), 8094–8105 (2008). 18545522 10.1364/oe.16.008094

[b8] Kildishev A. V. & Narimanov E. E. Impedance-matched hyperlens. Opt. Lett. 32(23), 3432–3434 (2007). 18059957 10.1364/ol.32.003432

[b9] Xu Y., Du S., Gao L. & Chen H. Overlapped illusion optics: a perfect lens brings a brighter feature. New J. Phys. 13, 023010 (2011).

[b10] Luo Y., Zhu S., Liu Y., Liu Z. & Fang S. Spatial power combiner using a planar lens array. J. Mod. Opt. 60, 906–914 (2013).

[b11] Li J. J. *et al.* Overlapped optics induced perfect coherent effects. Sci. Rep. 3, 3569 (2013). 24356577 10.1038/srep03569 PMC6506450

[b12] Luo Y. & Zhu S. Z. A power combiner and multisource co-beam reflector based on virtual shifing of the sources using negative index media. J. Opt. 14, 105102 (2012).

[b13] Zhang X. F. & Jiang C. Overlapped optics, illusion optics, and an external cloak based on shifting media. J. Opt. Soc. Am. B 28, 1994–2000 (2011).

[b14] Chen H., Xu Y., Li H. & Tomáš T. Playing the tricks of numbers of light sources. New J. Phys. 15, 093034 (2013).

[b15] Lai Y., Zheng H., Zhang Z. & Chan C. T. Manipulating sources using transformation optics with ‘folded geometry’. J. Opt. 13, 024009 (2011).

[b16] Lai Y. *et al.* Illusion Optics: The Optical Transformation of an Object into Another Object. Phys. Rev. Lett. 102, 253902 (2009). 19659076 10.1103/PhysRevLett.102.253902

[b17] Pendry J. B. Negative refraction makes a perfect lens. Phys. Rev. Lett. 85, 3966 (2000). 11041972 10.1103/PhysRevLett.85.3966

[b18] He Q., Xiao S., Li X. & Zhou L. Optic-null medium: realization and applications. Opt. Express 21, 28948–28959 (2013). 24514409 10.1364/OE.21.028948

[b19] Sadeghi M. M., Li S., Xu L., Hou B. & Chen H. Transformation optics with Fabry-Pérot resonances. Sci. Rep. 5, 8680 (2015). 10.1038/srep08680 PMC4345524 25726924

[b20] Sun F. & He S. Extending the scanning angle of a phased array antenna by using a null-space medium. Sci. Rep. 4, 6832 (2014). 10.1038/srep06832 PMC4213772 25355198

[b21] Sun F. & He S. Surface transformation with homogenous optic-null medium. Prog. Electromagn. Res. 151, 169–173 (2015).

[b22] Sun F. & He S. Optical Surface Transformation: Changing the optical surface by homogeneous optic-null medium at will. Sci. Rep. 5, 16032 (2015). 26515406 10.1038/srep16032 PMC4626842

[b23] Xu S. *et al.* Realization of deep subwavelength resolution with singular media. Sci. Rep. 4, 5212 (2014). 24909738 10.1038/srep05212 PMC4048882

[b24] Wang W. *et al.* Design of oblate cylindrical perfect lens using coordinate transformation. Opt. Express 16, 8094–8105 (2008). 18545522 10.1364/oe.16.008094

[b25] Navau C., Prat-Camps J., Romero-Isart O., Cirac J. I. & Sanchez A. Long-distance transfer and routing of static magnetic fields. Phys. Rev. Lett. 112, 253901 (2014). 25014816 10.1103/PhysRevLett.112.253901

[b26] Sadeghi M. M., Nadgaran H. & Chen H. Perfect field concentrator using zero index metamaterials and perfect electric conductors. Front. Phys. 9, 90–93 (2014).

[b27] Sun F. & He S. Transformation inside a Null-Space Region and a DC Magnetic Funnel for Achieving an Enhanced Magnetic Flux with a Large Gradient Prog. Electromagn. Res. 146, 143–153 (2014).

[b28] Zhang X. F. *et al.* Illusion induced overlapped optics. Opt. Express 22, 582–592 (2014). 24515019 10.1364/OE.22.000582

[b29] Yang J. J., Francescato Y., Maier S. A., Mao F. & Huang M. Mu and epsilon near zero metamaterials for perfect coherence and new antenna designs. Opt. Express 22, 9107–9114 (2014). 24787800 10.1364/OE.22.009107

